# Impact of Smoking on the Risk of Pancreatitis: A Systematic Review and Meta-Analysis

**DOI:** 10.1371/journal.pone.0124075

**Published:** 2015-04-16

**Authors:** Xiaohua Ye, Guangrong Lu, Jiaping Huai, Jin Ding

**Affiliations:** 1 Department of Gastroenterology and Hepatology, Jinhua Municipal Central Hospital, Jinhua Hospital of Zhejiang University, Jinhua, Zhejiang, P.R. China; 2 Department of Gastroenterology and Hepatology, the Second Affiliated Hospital of Wenzhou Medical University, Wenzhou, Zhejiang, P.R. China; 3 Department of Critical Care Medicine, Jinhua Municipal Central Hospital, Jinhua Hospital of Zhejiang University, Jinhua, Zhejiang, P.R. China; National Cancer Center, JAPAN

## Abstract

**Background and objective:**

Cigarette smoking may increase the risk of developing pancreatic cancer, although its impact on pancreatitis has only been discerned in recent years. However, the results of previous studies differ. We performed a meta-analysis to provide a quantitative pooled risk estimate of the association of cigarette smoking with pancreatitis.

**Method:**

A literature search of the MEDLINE and Embase databases was conducted, and studies were selected that investigated the association of cigarette smoking with pancreatitis. Summary relative risks (RRs) with 95% confidence intervals (CIs) were pooled using a random-effects model.

**Results:**

Twenty-two studies were included. The summary RRs (95% CI) associated with ever, current and former smokers for acute and chronic pancreatitis (AP/CP) were as follows: 1.51 (1.10, 2.07)/3.00 (1.46, 6.17), 1.42 (1.08, 1.87)/2.72 (1.74, 4.24), and 1.22 (0.99, 1.52)/1.27 (1.00, 1.62), respectively. Moreover, studies that analyzed both AP and CP were also summarized: 1.73 (1.18, 2.54) for ever smokers, 1.67 (1.03, 2.68) for current smokers and 1.56 (1.16, 2.11) for former smokers, respectively. There was no evidence of publication bias across the studies.

**Conclusion:**

The evidence suggests a positive association of cigarette smoking with the development of pancreatitis. It is possible that smoking cessation may be a useful strategy for the management of pancreatitis.

## Introduction

The incidence of acute pancreatitis (AP) and chronic pancreatitis (CP) has increased in recent decades [1–3]. AP is defined as an acute inflammatory process of the pancreas and is a frequent cause of hospitalization in the USA [4]. The premature activation of proenzymes within the pancreas is generally accepted as the cause of AP. Cholelithiasis, excessive alcohol intake, hyperlipidemia, pancreatic trauma, infections, and medications are the well-documented risk factors [1–3]. Abundant data indicate that AP progresses to recurrent AP and then to CP in a disease continuum [4]. Although the incidence of CP is lower, patients with CP have a lower quality of life and shorter lifespan than the general population [4].

Cigarette smoking is a well-established risk factor for the development of pancreatic cancer [5]. However, the reported correlations between smoking and pancreatitis risk are inconsistent [6–27]: some studies showed a positive correlation [6–8,11–15,17,19–24,27], whereas others failed to demonstrate such an association [9,10,16,18,25,26]. This lack of consistency across studies may be due to differences in study populations, differences in methodology or exposure definitions, variations in the quantity of cigarettes consumed, or a shortage of data on confounding factors. Additionally, alcohol intake may confound the correlation between smoking and pancreatitis because individuals who drink alcohol also often smoke and vice versa. Several experimental studies have shown that smoking induces pathological and functional changes in the exocrine pancreas. Nicotine induces damage through signal transduction pathways in pancreatic acinar cells, leading to elevated levels of intracellular calcium release and/or impaired pancreatic blood flow. Moreover, nicotine also alters gene expression in the exocrine pancreas, which affects the ratio of trypsinogen to its endogenous inhibitor [28–30]. One recent meta-analysis summarized the results from 12 studies and confirmed the detrimental effects of smoking on the pancreas [31]. The risk of CP was more than 2-fold greater among current smokers compared with never smokers. Also, a dose-response effect of smoking on CP risk was confirmed. However, this study was limited by the inappropriate inclusion of patients with AP and studies using unspecified definitions of CP, which may have biased the conclusions.

The knowledge that cigarette smoking is a potential risk factor for pancreatitis would allow preventive measures for high-risk patients and could lead to new intervention strategies. Therefore, we performed a meta-analysis to investigate the correlation between smoking and pancreatitis using recently published studies.

## Methods

### Search strategy and data extraction

We conducted this meta-analysis by following the Meta-Analysis of Observational Studies in Epidemiology guidelines [32]. Two investigators (Ye XH and Huai JP) independently performed a search of MEDLINE (from 1 January 1966 to 31 July 2014) and Embase (from 1 January 1974 to 31 July 2014) to identify potentially relevant articles. The following search terms were used: smoking (“tobacco”, “cigarette”, “smoke”, “smoking”), and pancreatitis (“acute pancreatitis”, “chronic pancreatitis”, “pancreatitis”). Manual searches of the bibliographies from these potential articles were also conducted to identify additional studies relevant to the review. Only the citations from English-language literature that met the following inclusion criteria were included: (1) case-control or cohort design and published in manuscript form; (2) smoking included as an exposure of interest; (3) pancreatitis included as an outcome of interest; and (4) studies reported relative risks (RRs) or odds ratios (ORs) and their corresponding 95% confidence intervals (CI) of pancreatitis for different smoking categories. If multiple reports based on the same population were retrieved, the most informative study was selected. Studies were excluded if: (1) the definition of pancreatitis was not specified; (2) data were not meta-analyzable (such as letters, reviews, practice guidelines, editorials, case reports and consensus statements); or (3) duplicate reports.

Data were independently extracted by two authors (Ye XH and Huai JP) using a standardized data collection form. For each eligible study, the following data were extracted: first author’s last name, publication year, location of the study population, study design, number of subjects, smoking categories (ever, current and former), variables adjusted in the analysis, and RR with corresponding 95% CI for each category of smoking exposure. If an RR was not reported, it was calculated using the original data (number of cases and control subjects exposed to smoking) from the study. Any disagreement was resolved by consensus.

### Assessment of study quality

The well-established, validated Newcastle-Ottawa scale (NOS) was used for assessing the quality of the included studies [33]. It allocates a maximum of 9 points to each of three categories: (1) patient selection (three items); (2) comparability of the two study arms (two items); and (3) assessment of outcome (two items). Studies with 7–9 points were considered of high quality, studies with 5–6 points were considered of moderate quality, and studies with 0–4 points were considered of poor quality [34]. The NOS score was assessed independently by two reviewers. Discrepancies were resolved through discussions between the reviewers.

### Statistical analyses

Different measures of risk were included in this meta-analysis: case-control studies (OR) and cohort studies (RR). Because clinical heterogeneity existed among the definitions of smoking, we calculated the summary RRs with their corresponding 95% CIs using a random-effects model [35]. When more than one category of smoking fell into the same level, we combined the corresponding RRs using the method of Hamling et al. [36]. If studies reported separate RRs for males and females, we calculated the pooled RR and its corresponding 95% CI. We conducted further analyses stratified by study design, geographic region, source, gender, etiology of pancreatitis, cigarette consumption, and adjustment for alcohol intake.

A dose-response meta-analysis of the correlation between smoking and the risk of pancreatitis was also performed using a generalized least-squares trend estimation analysis (GLST) [37,38]. To determine the dose-response relationship, we included studies that reported at least three categories representing the levels of cigarette smoking and provided the number of cases and participants, the adjusted RR, and the corresponding 95% CI. The midpoint of each exposure category was assigned to each corresponding risk estimate. When the highest category was open-ended, we assumed that it had the same amplitude as the preceding category. If the lowest category was open-ended, then the lowest boundary was considered as zero.

Heterogeneity was evaluated using the Q-statistic and quantified using *I* ^*2*^ [39]. For the *Q* test, *P* < 0.10 was considered to imply statistical heterogeneity. *I* ^*2*^ is the proportion of total variation contributed by between-study variation. Publication bias was evaluated using Begg’s funnel plot and Egger’s test [40,41]. All statistical analyses were performed using STATA software (version 12.0; College Station, Texas, USA).

## Results

### Study identification and characteristics

**Fig 1:**
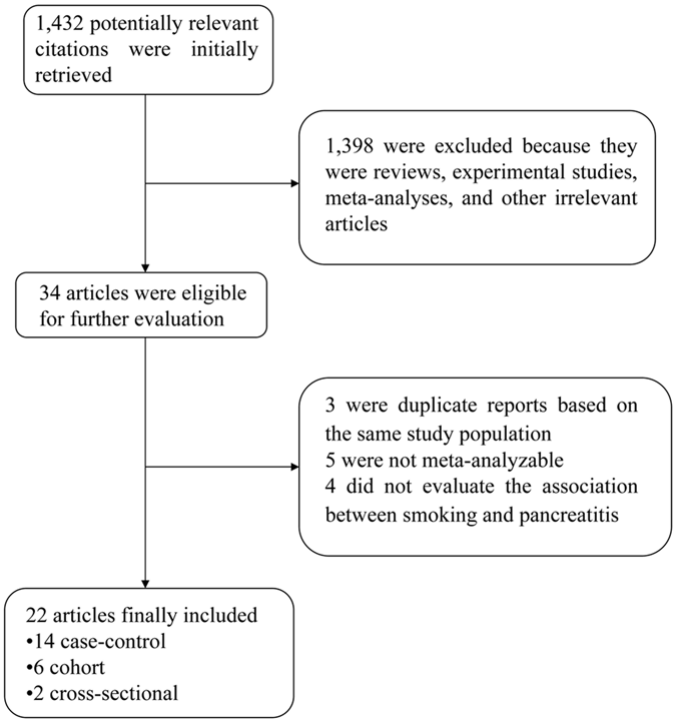
Flow chart of the study selection.

Our initial literature search retrieved 1,432 articles, and 1,398 were excluded after an inspection of the titles or abstracts revealed that they were reviews, experimental studies, meta-analyses or other irrelevant articles. Of the remaining 34 articles, 12 articles were subsequently excluded from the meta-analysis. Three were duplicate reports based on the same study population, five were not meta-analyzable, and four did not evaluate the correlation between smoking and pancreatitis. As a result, 22 studies [6–27] were identified in this meta-analysis (Fig 1).

The characteristics of the 22 included studies are listed in Table 1. The studies were published between 1982 and 2014 and were performed in 10 countries. Of the 22 studies, 9 were conducted in the USA, 9 in Europe, 3 in Asia, and 1 in Australia. Fourteen studies recruited both male and female participants, and 5 studies included only men. Three studies did not report the number of male and female patients [17,21,26]. The number of patients in each study ranged from 36 to 1,000 and included 4,831 cases with pancreatitis. Adjustments were made for potential confounding factors in 13 of the 22 studies. Most of the included studies were of moderate-to-high quality (Table 2).

**Table 1:** Characteristics of studies included in the meta-analysis.

Study	Region	Studydesign	Source	No. of cases	Gender (cases) (M/F)	No. of controls	Gender (controls) (M/F)	Smoking categories	Adjustments	Type of pancreatitis
Yen et al. 1982 [6]	US	Case-control	Hospital	98	53/45	550	218/233	E, C, F	Age, alcohol	Chronic, recurrent or relapsing
Lowenfels et al. 1987 [7]	US	Case-control	Alcoholic cirrhotics	54	45/9	56	26/30	E	None	Alcoholic
Bourliere et al. 1991 [8]	France	Case-control	Population	132	132/0	167	167/0	E	None	Chronic, alcoholic
Haber et al. 1993 [9]	Australia	Case-control	Alcoholics	52	44/8	47	39/8	E, C, F	None	Chronic, alcoholic
Lévy et al. 1995 [10]	France	Case-control	Alcoholics	56	56/0	50	50/0	E	None	Chronic, alcoholic
Talamini et al. 1996 [11]	Italy	Case-control	Alcoholics	463	463/0	265	265/0	E	Alcohol	Chronic, acute, alcoholic
Lin et al. 2000 [12]	Japan	Case-control	Hospital	91	91/0	175	175/0	E, C, F	BMI, education level, alcohol	Chronic
Blomgren et al. 2002 [13]	Sweden	Case-control	Population	462	260/202	1781	865/916	C	NR	Acute, alcoholic, gallstone, and other
Morton et al. 2004 [14]	US	Cohort	Population	403	197/206	128934	56926/72008	E, C, F	Age, sex, race, BMI, education, alcohol	Chronic, acute, alcoholic, gallstone, idiopathic
Rothenbacher et al. 2005 [15]	Germany	Case-control	Population	47	23/24	762	314/448	C, F	Age, ACE inhibitor intake	Chronic, severe exocrine pancreatic insufficiency
Yadav et al. 2007 [16]	US	Case-control	Alcoholics	39	39/0	1363	1363/0	E, C, F	None	Chronic, acute, alcoholic
Lindkvist et al. 2008 [17]	Sweden	Cohort	Population	179	NR	33263	22381/10882^1^	E, C, F	Age, sex, Mm-MAST category, BMI	Acute, alcoholic, gallstone,and other
Debenedet et al. 2009 [18]	US	Case-control	Hospital	123	32/91	248	65/183	E, C, F	Age < 60 years, sex	acute, PEP
Tolstrup et al. 2009 [19]	Denmark	Cohort	Population	235	122/113	17905	8332/9573^1^	E, C, F	Sex, education, BMI, alcohol	Chronic, acute
Yadav et al. 2009 [20]	US	Case-control	Population	1000	488/512	695	249/446	E, C, F	Age, sex, BMI, alcohol	Chronic, acute, recurrent
Gonzalez-Perez et al. 2010 [21]	UK	Cohort	Population	419	NR	5000	NR	E, C, F	Age, sex, BMI, alcohol, ischemic heart diseases, medication use, gastrointestinal diseases	Acute
Law et al. 2010 [22]	US	Cross-sectional	Hospital	79	42/37	156	46/110	E, C, F	Age, sex, acute pancreatitis, alcohol, other factors	Chronic
Li et al. 2010 [23]	US	Case-control	Hospital	36	13/23	470	194/276	C	NR	Acute, PEP
Sadr-Azodi et al. 2012 [24]	Sweden	Cohort	Population	541	300/241	84667	45781/38886^1^	C, F	Age, sex, BMI, diabetes, educational level, monthly alcohol consumption	Acute, gallstone-related and non-gallstone-related
DiMagno et al. 2013 [25]	US	Case-control	Hospital	211	53/158	348	148/200	C, F	NR	Acute, PEP
Lin et al. 2014 [26]	Taiwan	Cohort	Population	66	NR	25404	8640/16764	E, C, F	Sex, alcohol, income, education, physical activity, biliary stone	Chronic, acute
Yang et al. 2014 [27]	China	Cross-sectional	Population	45	27/18	23249	11726/11523	E	NR	Acute

Abbreviations: *BMI* body mass index; *C* current; *E* ever; *F* former; *Mm-MAST* Malmö modification of the brief Michigan Alcoholism Screening Test; *NR* not reported; *PEP* post-endoscopic retrograde cholangiopancreatography pancreatitis

^1^Cohort number

**Table 2:** Methodological quality of included studies.

Author	Selection				Comparability		Assessment of outcome			NOSscore
	Ia	Ib	Ic	Id	IIa	IIb	IIIa	IIIb	IIIc	
Yen et al. 1982 [6]	*	*	*	*	*		*	*		7
Lowenfels et al. 1987 [7]	*	*	*				*	*	*	6
Bourliere et al. 1991 [8]	*	*	*	*	*	*		*	*	8
Haber et al. 1993 [9]	*	*		*	*	*		*	*	7
Lévy et al. 1995 [10]	*	*		*	*	*		*	*	7
Talamini et al. 1996 [11]		*		*	*	*		*	*	6
Lin et al. 2000 [12]	*	*		*	*	*		*	*	7
Blomgren et al. 2002 [13]	*	*	*	*			*	*	*	7
Morton et al. 2004 [14]	*	*	*	*	*	*	*	*	*	9
Rothenbacher et al. 2005 [15]		*	*		*	*	*	*	*	7
Yadav et al. 2007 [16]	*	*		*	*	*		*	*	7
Lindkvist et al. 2008 [17]		*		*	*	*	*	*	*	7
Debenedet et al. 2009 [18]	*	*		*	*	*		*	*	7
Tolstrup et al. 2009 [19]		*	*	*	*	*	*	*	*	8
Yadav et al. 2009 [20]	*	*		*	*	*		*	*	7
Gonzalez-Perez et al. 2010 [21]	*	*	*	*	*	*	*	*	*	9
Law et al. 2010 [22]	*	*		*	*	*		*	*	7
Li et al. 2010 [23]	*	*		*	*	*	*	*	*	8
Sadr-Azodi et al. 2012 [24]		*	*	*	*	*	*	*	*	8
DiMagno et al. 2013 [25]		*		*	*	*		*	*	6
Lin et al. 2014 [26]	*	*	*	*	*	*	*	*	*	9
Yang et al. 2014 [27]	*	*	*	*	*	*	*	*	*	9

For case-control studies: (Ia) cases with independent validation; (Ib) consecutive or representative cases; (Ic) community controls; (Id) controls with no history of pancreatitis; (IIa) study controls were comparable for age and gender; (IIb) study controls were comparable for all additional factor(s) reported; (IIIa) the same method of ascertainment was used for cases and controls; (IIIb) assessment of exposure was from a secure record; and (IIIc) the non-response rate was similar for both groups. For cohort studies: (Ia) the exposed cohort was representative of the population; (Ib) the non-exposed cohort was drawn from the same population; (Ic) the exposure ascertainment was from secure records or a structured interview; (Id) pancreatitis was not present at start of the study; (IIa) the cohorts were comparable for age and gender; (IIb) the cohorts were comparable for all additional factor(s) reported; (IIIa) cases were assessed from a secure record; (IIIb) follow-up was long enough for pancreatitis to occur; and (IIIc) follow-up was complete. NOS: Newcastle-Ottawa score.

### Smoking and AP

**Fig 2:**
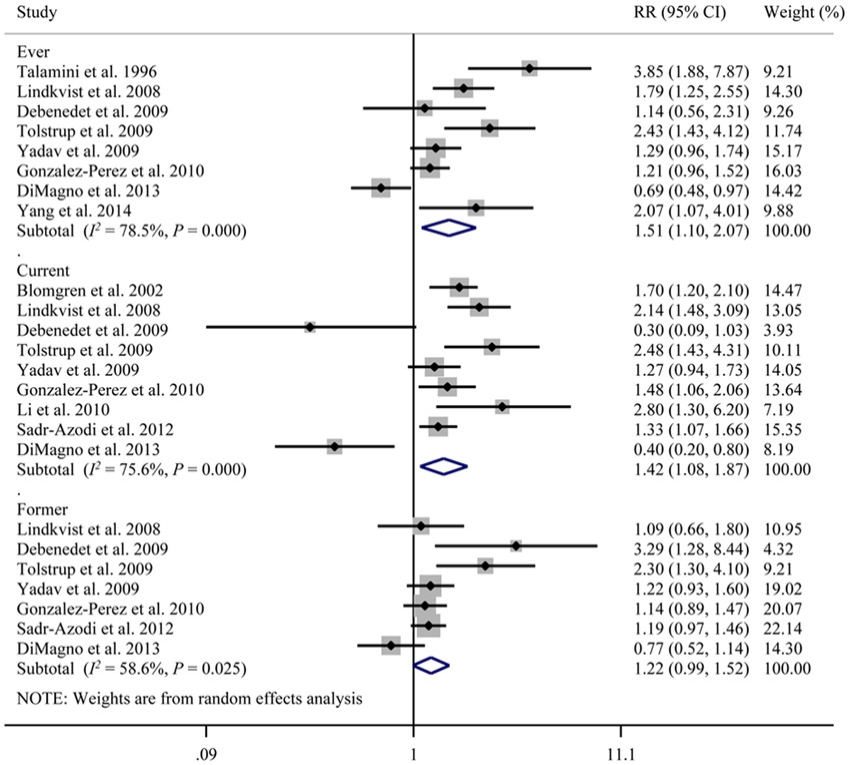
Cigarette smoking and the risk of acute pancreatitis. CI, confidence interval; RR, relative risk.

Eleven studies that included 2,703 patients investigated the correlation between smoking and the development of AP (Fig 2) [11,13,17–21,23–25,27]. Study data were collected from 1996 to 2014. The meta-analysis of ever (*versus* never) smokers included eight studies [11,17–21,25,27]. The summary RR associated with ever smoking was 1.51 (95% CI: 1.10, 2.07), and there was significant heterogeneity among studies (Q = 32.50, *P* < 0.001, *I* ^*2*^ = 78.5%). Nine studies analyzed current (*versus* never) smokers [13,17–21,23–25], and the combined results indicated that current smokers were also more likely to develop AP (RR = 1.42, 95% CI: 1.08, 1.87). The heterogeneity was significant among the studies on current smokers (Q = 32.84, *P* < 0.001, *I* ^*2*^ = 75.6%). When the seven studies on former (*versus* never) smokers were combined [11,17–20,24,25], the correlation with AP was marginal (RR = 1.22, 95% CI: 0.99, 1.52), and significant heterogeneity was detected across the studies (Q = 14.49, *P* = 0.025, *I* ^*2*^ = 58.6%).

### Smoking and CP

**Fig 3:**
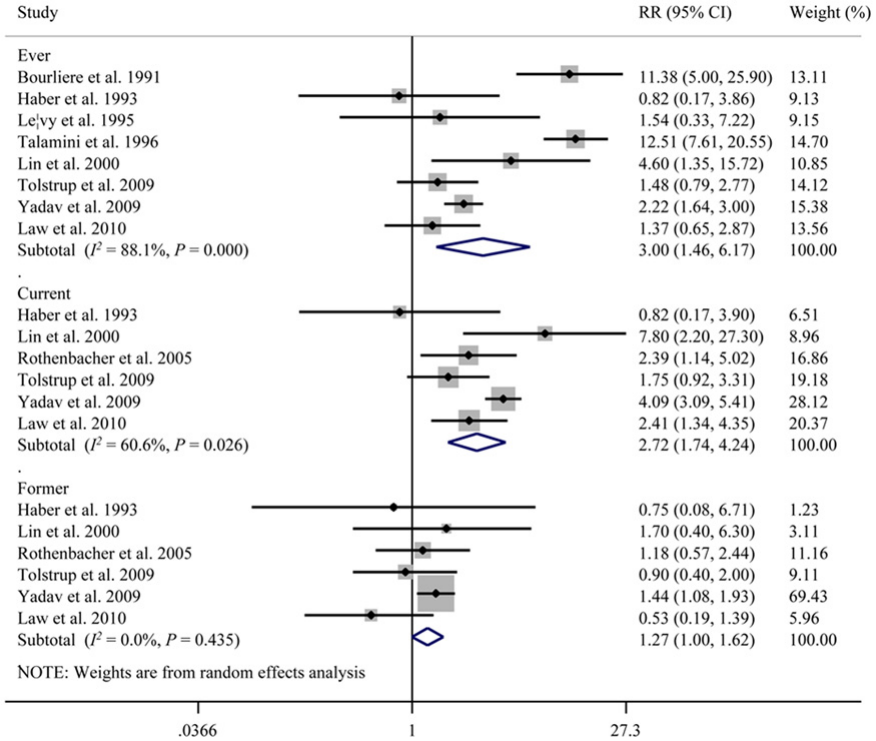
Cigarette smoking and the risk of chronic pancreatitis. CI, confidence interval; RR, relative risk.

Nine studies that included 1,490 patients investigated the correlation between smoking and the development of CP (Fig 3) [8–12,15,19,20,22]. Study data were collected from 1991 to 2010. The meta-analysis of eight studies on ever (*versus* never) smokers revealed a positive correlation between smoking and developing CP (RR = 3.00, 95% CI: 1.46, 6.17); however, significant heterogeneity was detected among the studies (Q = 58.65, *P* < 0.001, *I* ^*2*^ = 88.1%). Six studies reported RRs on the development of CP for current and former smokers [9,12,15,19,20,22]. The summary RRs were 2.72 (95% CI: 1.74, 4.24) for current smokers and 1.27 (95% CI: 1.00, 1.62) for former smokers. Significant heterogeneity was found for the current smokers (Q = 12.70, *P* = 0.026, *I* ^*2*^ = 60.6%), whereas no significant heterogeneity was detected for the former smokers (Q = 6.56, *P* = 0.435, *I* ^*2*^ = 0.0%).

### Smoking and pancreatitis

**Fig 4:**
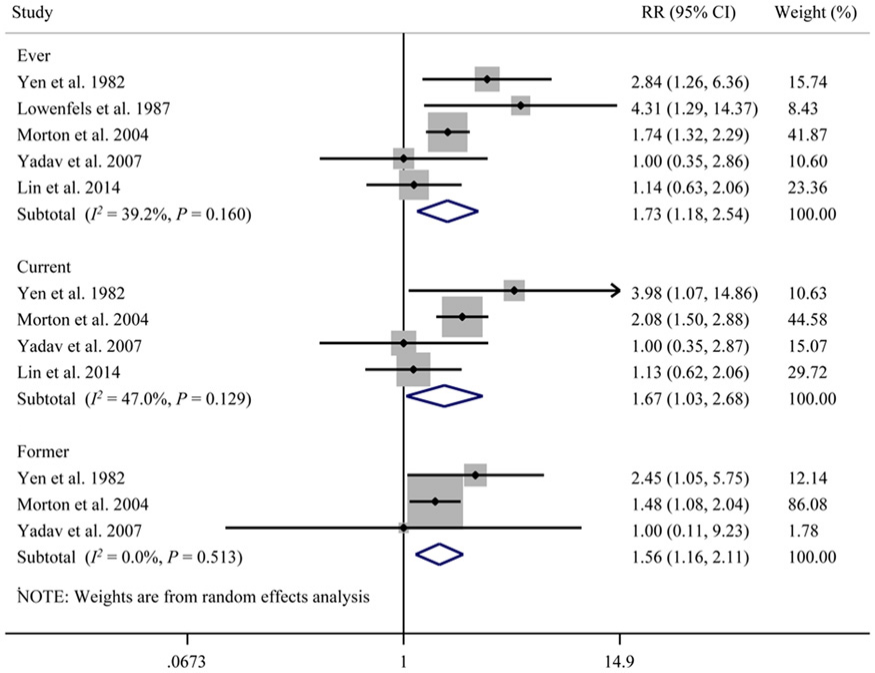
Cigarette smoking and the risk of pancreatitis (acute and chronic combined into one single outcome). CI, confidence interval; RR, relative risk.

Because abundant evidence now indicates that AP and CP are manifestations of the same disease process [26], we also included studies that combined AP and CP into one single outcome (pancreatitis) for analysis. Five studies, which included 660 patients, investigated the correlation between smoking and the development of pancreatitis (Fig 4) [6,7,14,16,26]. Study data were collected from 1982 to 2014. The summary RRs of the five studies for ever (*versus* never) smokers was 1.73 (95% CI: 1.18, 2.54), and the heterogeneity was not significant (Q = 6.58, *P* = 0.16, *I* ^*2*^ = 39.2%). The pooling of four studies [6,14,16,26] that evaluated current (*versus* never) smokers also revealed a correlation between current smoking and pancreatitis (RR = 1.67, 95% CI: 1.03, 2.68). There was no significant heterogeneity among studies (Q = 5.66, *P* = 0.129, *I* ^*2*^ = 47.0%). The meta-analytical results for three studies [6,14,16] associated with former (*versus* never) smokers yielded an RR of 1.56 (95% CI: 1.16, 2.11). No significant heterogeneity was detected across the studies (Q = 1.34, *P* = 0.513, *I* ^*2*^ = 0.0%).

### Dose-response analysis

**Fig 5:**
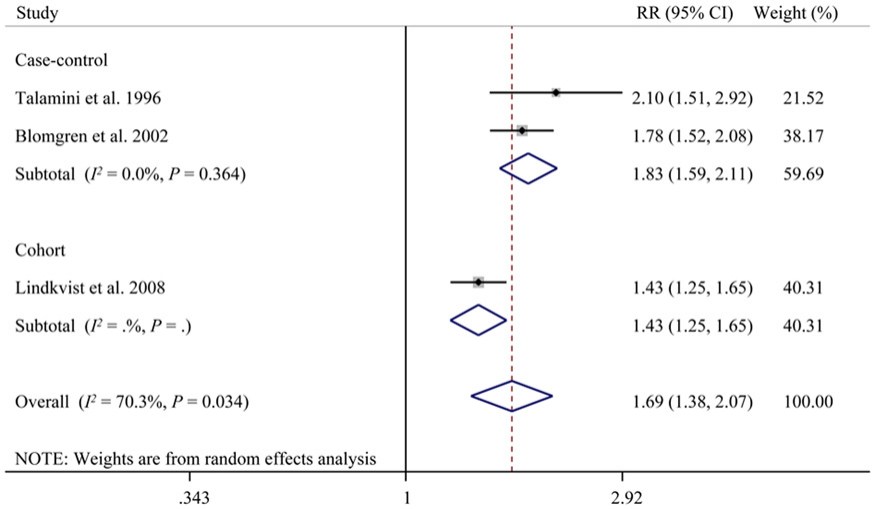
Dose-response meta-analysis of cigarette smoking and the risk of acute pancreatitis. CI, confidence interval; RR, relative risk; RR indicates the risk of acute pancreatitis per 10 cigarettes increment in amount of smoking.

Three studies (two case-controls and one cohort) were eligible, i.e., they contained the required data for a dose-response analysis [11,13,17]. As shown in Fig 5, the pooled results indicated that a 10 cigarettes/day increment was significantly associated with a 69% increase in the risk of AP (RR = 1.69, 95% CI: 1.37, 2.07). Significant heterogeneity was found between studies (Q = 6.74, *P* = 0.034, *I* ^*2*^ = 70.3%); however, the heterogeneity became unremarkable when the cohort study was excluded (Q = 0.82, *P* = 0.364, *I* ^*2*^ = 0.0%).

### Subgroup analysis

The summary RRs of the subgroup analyses based on study characteristics are presented in Table 3. For AP, the meta-analysis results were non-significant in some strata when data were stratified by region (USA), design (case-control), source (hospital), and etiology (post-endoscopic retrograde cholangiopancreatography pancreatitis, PEP). Moreover, the subgroup analysis based on etiology (non-PEP/PEP) reduced the heterogeneity of the correlation between smoking and AP (S1 Fig). However, the results did not differ significantly in the CP and pancreatitis groups.

**Table 3:** Subgroup analyses for the correlation between smoking and pancreatitis.

Subgroups	Ever					Current					Former				
	No. of studies	RR (95% CI)	Tests for heterogeneity			No. of studies	RR (95% CI)	Tests for heterogeneity			No. of studies	RR (95% CI)	Tests for heterogeneity		
			Q	*P*	*I* ^*2*^ (%)			Q	*P*	*I* ^*2*^ (%)			Q	*P*	*I* ^*2*^ (%)
*Acute pancreatitis*															
Geographical region															
Europe	4	1.95 (1.25, 3.05)	14.04	0.003	78.6	5	1.67 (1.36, 2.05)	7.94	0.094	49.6	4	1.25 (1.01, 1.56)	5.21	0.157	42.4
US	3	0.99 (0.62, 1.57)	7.55	0.023	73.5	4	0.87 (0.37, 2.03)	18.90	0.000	84.1	3	1.25 (0.72, 2.18)	8.86	0.012	77.4
Study design															
Case-control	4	1.33 (0.73, 2.41)	19.94	0.000	85.0	5	1.06 (0.61, 1.85)	23.78	0.000	83.2	3	1.25 (0.72, 2.18)	5.21	0.157	42.4
Cohort	3	1.65 (1.11, 2.45)	7.36	0.025	72.5	4	1.69 (1.29, 2.23)	7.67	0.053	60.9	4	1.25 (1.01, 1.56)	8.86	0.012	77.4
Source															
Population	5	1.56 (1.22, 2.00)	9.01	0.061	55.6	6	1.59 (1.33, 1.91)	9.69	0.085	48.3	5	1.23 (1.05, 1.43)	5.21	0.266	23.2
Hospital	2	0.80 (0.51, 1.26)	1.57	0.221	36.1	3	0.72 (0.17, 2.99)	16.21	0.000	87.7	2	1.49 (0.36, 6.15)	7.76	0.005	87.1
Gender															
Male	2	2.16 (0.76, 6.11)	6.64	0.01	84.9	2	1.92 (1.48, 2.50)	0.52	0.470	0.0	1	1.19 (0.78, 1.80)	—	—	—
Female	1	1.11 (0.82, 1.52)	—	—	—	2	1.25 (0.97, 2.13)	1.08	0.299	7.2	1	1.15 (0.79, 1.67)	—	—	—
Etiology															
non-PEP	6	1.76 (1.31, 2.36)	15.98	0.007	68.7	6	1.59 (1.33, 1.91)	9.68	0.085	48.3	5	1.23 (1.05, 1.43)	5.21	0.266	23.2
PEP	2	0.80 (0.51, 1.26)	1.57	0.211	36.1	3	0.72 (0.17, 2.99)	16.21	0.000	87.7	2	1.49 (0.36, 6.15)	7.76	0.005	87.1
Amount															
< 1 pack/d	2	1.54 (0.58, 4.09)	5.20	0.023	80.8	2	1.69 (1.37, 2.07)	0.19	0.661	0.0	—	—	—	—	—
≥ 1 pack/d	2	2.97 (0.73, 11.99)	9.63	0.002	89.6	2	3.35 (2.38, 4.73)	0.47	0.495	0.0	—	—	—	—	—
Adjusted for alcohol use	4	1.76 (1.19, 2.60)	12.65	0.005	76.3	3	1.55 (1.17, 2.07)	4.25	0.120	52.9	3	1.30 (0.99, 1.72)	5.01	0.081	60.1
*Chronic pancreatitis*															
Geographical region															
Europe	4	4.53 (1.30, 15.76)	32.77	0.000	90.8	2	2.00 (1.23, 3.24)	0.39	0.530	0.0	2	1.04 (0.61, 1.79)	0.24	0.624	0.0
USA	2	1.97 (1.31, 2.96)	1.41	0.236	28.9	2	3.35 (2.03, 5.53)	2.52	0.112	60.3	2	0.98 (0.38, 2.56)	3.59	0.058	72.1
Study design															
Case-control	6	3.92 (1.56, 9.84)	46.26	0.000	89.2	4	3.23 (1.76, 5.92)	6.73	0.081	55.4	4	1.40 (1.08, 1.82)	0.64	0.886	0.0
Cohort	1	1.48 (0.79, 2.77)	—	—	—	1	1.75 (0.92, 3.31)	—	—	—	1	0.90 (0.40, 2.01)	—	—	—
Cross-sectional	1	1.37 (0.65, 2.87)	—	—	—	1	2.41 (1.34, 4.34)	—	—	—	1	0.53 (0.20, 1.43)	—	—	—
Source															
Population	3	3.16 (1.24, 8.02)	16.28	0.000	87.7	3	2.74 (1.55, 4.86)	6.70	0.035	70.2	3	1.34 (1.04, 1.93)	1.31	0.520	0.0
Hospital	2	2.26 (0.70, 7.31)	2.75	0.095	63.7	2	3.78 (1.23, 11.57)	2.74	0.098	63.5	2	0.86 (0.28, 2.64)	1.81	0.179	44.6
Alcoholic	3	2.80 (0.41, 19.07)	15.74	0.000	87.3	1	0.82 (0.17, 3.90)	—	—	—	1	0.75 (0.08, 6.70)	—	—	—
Gender															
Male	5	5.62 (2.58, 12.25)	19.98	0.001	80.0	2	7.15 (4.60, 11.11)	0.02	0.884	0.0	2	1.53 (0.98, 2.40)	0.02	0.876	0.0
Female	1	1.79 (1.22, 2.64)	—	—	—	1	2.53 (1.76, 3.64)	—	—	—	1	1.26 (0.84, 1.88)	—	—	—
Amount															
< 1 pack/d	2	5.08 (0.89, 29.04)	33.17	0.000	97.0	1	14.7 (3.10, 69.9)	—	—	—	—	—	—	—	—
≥ 1 pack/d	2	5.89 (1.43, 24.33)	17.19	0.000	94.2	1	6.31 (1.75, 22.70)	—	—	—	—	—	—	—	—
Adjusted for alcohol use	6	2.71 (1.15, 6.40)	44.73	0.000	88.8	4	2.83 (1.38, 5.83)	10.61	0.014	71.7	4	1.37 (1.05, 1.78)	1.56	0.669	0.0
*Pancreatitis*															
Geographical region															
Europe	—	—	—	—	—	—	—	—	—	—	—	—	—	—	—
USA	4	1.97 (1.27, 3.04)	4.47	0.215	32.9	3	1.97 (1.18, 3.30)	2.76	0.252	27.4	3	1.56 (1.16, 2.11)	1.34	0.513	0.0
Study design															
Case-control	3	2.28 (1.03, 5.03)	3.71	0.156	46.1	2	1.88 (0.49, 7.22)	2.57	0.109	61.1	2	2.19 (0.99, 4.85)	0.55	0.460	0.0
Cohort	2	1.53 (1.05, 2.24)	1.60	0.206	37.6	2	1.62 (0.90, 2.92)	3.08	0.079	67.5	1	1.48 (1.08, 2.04)	—	—	—
Source															
Population	2	1.53 (1.05, 2.24)	1.60	0.206	37.6	2	1.62 (0.90, 2.92)	3.08	0.079	67.5	1	1.48 (1.08, 2.04)	—	—	—
Hospital	1	2.84 (1.27, 6.36)	—	—	—	1	3.98 (1.06, 14.86)	—	—	—	1	2.45 (1.05, 5.76)	—	—	—
Alcoholic	2	2.01 (0.48, 8.39)	3.20	0.074	68.7	1	1.00 (0.35, 2.87)	—	—	—	1	1.00 (0.11, 9.24)	—	—	—
Gender															
Male	3	2.68 (0.85, 8.46)	4.77	0.092	58.1	2	2.82 (0.30, 26.60)	4.76	0.029	79.0	2	2.37 (0.60, 9.47)	0.95	0.33	0.0
Female	1	2.29 (1.00, 5.24)	—	—	—	1	2.44 (1.00, 6.00)	—	—	—	1	2.10 (0.80, 5.60)	—	—	—
Amount															
< 1 pack/d	—	—	—	—	—	2	2.37 (1.23, 4.57)	1.77	0.183	43.5	—	—	—	—	—
≥ 1 pack/d	—	—	—	—	—	2	2.98 (1.90, 4.67)	0.15	0.696	0.0	—	—	—	—	—
Adjusted for alcohol use	5	1.73 (1.18, 2.54)	6.58	0.160	39.2	4	1.67 (1.03, 2.68)	5.66	0.129	47.0	3	1.56 (1.16, 2.11)	1.34	0.513	0.0

Abbreviations: *RR* relative risk; *No*. number

When we limited the analysis to studies that controlled for alcohol consumption or used patients with alcoholism as a control group, the summary RRs remained significant in the AP, CP, and pancreatitis groups.

We considered that dose differences might contribute to the observed heterogeneity of the results. Thus, another analysis was performed using categories that defined cigarette consumption per day. Seven studies reported that the development of AP [11, 13, 17, 20], CP [11, 12, 20] or pancreatitis (AP and CP combined) [6, 14] correlated with cigarette consumption. For AP, there was no heterogeneity detected for patients who smoked 1–10 or 11–20 cigarettes per day, whereas severe heterogeneity was found for those who smoked ≥1 pack (1 pack = 20 cigarettes) per day (Q = 18.34, *P* < 0.001, *I* ^*2*^ = 83.6%; Table 4). There was also a significant dose-response relation in current smokers: the RR was 1.69 (95% CI: 1.37, 2.07) for patients smoking <1 pack per day and 3.35 (95% CI: 2.38, 4.73) for patients smoking ≥1 pack per day.

**Table 4:** Relative risk (RR) and 95% confidence interval (95% CI) for categories based on daily cigarette consumption.

Amount of cigarettes	No. of studies	RR (95% CI)	Tests for heterogeneity		
			Q	*P*	*I* ^*2*^ (%)
1–10	2	1.12 (0.82, 1.52)	0.17	0.682	0.0
11–20	2	2.70 (2.02, 3.60)	0.51	0.477	0.0
>20	4	3.08 (.169, 5.62)	18.34	0.000	83.6

Abbreviations: *No*. number.

The subgroup analysis based on gender indicated a significant correlation between smoking and CP for both sexes. The significantly greater RRs for male patients with CP were 5.62 (95% CI: 2.58, 12.25) for ever smokers and 7.15 (95% CI: 4.60, 11.11) for current smokers. The RRs for female patients were 1.79 (95% CI: 1.22, 2.64) and 2.53 (95% CI: 1.76, 3.64) for ever and current smokers, respectively.

### Publication bias

**Fig 6:**
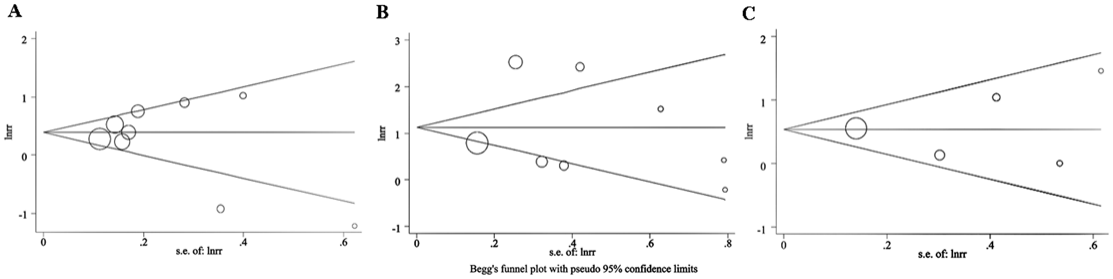
Begg’s funnel plot with pseudo 95% confidence limits showing the symmetrical distribution of the included studies. Begg’s funnel plot for acute pancreatitis (A), for chronic pancreatitis (B), and for pancreatitis (C; acute and chronic combined into one single outcome).

There was no evidence for a significant publication bias: for AP, *P* = 1.000 using the Begg’s test and *P* = 0.577 using the Egger’s test; for CP, *P* = 0.621 using the Begg’s test and *P* = 0.989 using the Egger’s test; for pancreatitis, *P* = 0.327 using the Begg’s test and *P* = 0.782 using the Egger’s test. Therefore, the summary estimates were not substantively affected by publication bias (Fig 6).

## Discussion

To our knowledge, this systematic review and meta-analysis represents the first attempt to summarize recent studies investigating the impact of cigarette smoking on the development of pancreatitis. We included 22 studies that comprised 4,831 cases of pancreatitis. Our main finding was that both ever and current smokers had higher risks of developing pancreatitis compared with never smokers. Moreover, this causative relationship was verified by the reduced or eliminated risk after smoking cessation. In comparison with current smokers, the risk of pancreatitis decreased for those who were former smokers. Also, a significant correlation between smoking and CP was observed for both sexes, whereas the trend of developing CP was more pronounced for males. This finding raises the possibility of an interaction between gender and risk. Moreover, cigarette smoking was found to be associated with AP development in a dose-response manner. The likelihood of developing AP was increased in participants who smoked ≥1 pack per day over those who smoked <1 pack per day. Additionally, each increment of 10 cigarettes/day added an additional 69% risk of developing AP (Table 3 and Fig 5).

Our results have substantial clinical and public health implications. To date, the number of habitual smokers is increasing despite numerous anti-smoking campaigns [42]. Also, cigarette smoking affects the age at diagnosis and exacerbates the disease. It may also accelerate the progression towards cancer for patients with hereditary pancreatitis who are at an increased risk of developing pancreatic carcinomas [43]. The awareness of cigarette smoking as a potential risk factor for developing pancreatitis would allow preventive measures for high-risk patients and would support the proactive implementation of preventive strategies. Therefore, early education on the benefits of smoking cessation should be the responsibility of clinical physicians who have patients with pancreatitis. Moreover, physicians who realize the risk of pancreatitis for smokers will be able to help patients and their families understand the potential benefits of smoking cessation on outcome.

An important attribute of this study is that the quantitative risk assessment for pancreatitis was controlled for alcohol consumption. Nicotine can induce pancreatic damage and upregulate intracellular calcium release and/or impair pancreatic blood flow. Additionally, gene expression levels in the exocrine pancreas are altered by nicotine absorption, thus affecting the ratio of trypsinogen to its endogenous inhibitor [28–30]. Although previous experimental studies have demonstrated that smoking can lead to pathological and functional changes in the exocrine pancreas, there is still an argument against the role of cigarette smoking in pancreatitis development given that smokers also often consume alcohol [9,31,44]. Indeed, the role of smoking may be attenuated if alcohol consumption acts as a primary risk factor in the incidence of pancreatitis. However, the RR for both AP and CP remained significant and varied little after we separately analyzed studies that reported the alcohol use–adjusted RR or employed data for patients with alcoholism only. Therefore, strong evidence has been provided for the role of smoking in the causation of pancreatitis.

Another major strength of our meta-analysis is that we used more restricted study inclusion criteria and reported RRs for AP, CP, and pancreatitis (AP and CP combined as a single outcome) separately. A previous meta-analysis of 12 studies that solely investigated the correlation between smoking and CP revealed a role for smoking in CP development [31]. However, this meta-analysis was limited by the inclusion of a study that included cases of AP and studies that did not differentiate between AP and CP [6,7,14,16]. These limitations could distort the correlation and might lead to biased conclusions. To avoid such limitations, we excluded these studies and updated information regarding the correlation between smoking and CP, including nine studies of CP.

There was significant heterogeneity among certain results. The included studies were heterogeneous according to study region, design, source, etiology, and duration of follow-up. We used a random-effects model, which assumed that the true effects were normally distributed, and more weight was assigned to small-sized studies compared with in the fixed-effects model [45]. Subgroup analyses were also conducted to address heterogeneity (Table 3). Subgroup analyses of AP based on etiology (PEP *versus* non-PEP) reduced the heterogeneity in all categories of cigarette smoking. Two of the PEP studies showed an inverse relationship compared with the non-PEP studies [18,25]. Nicotine may activate the nicotinic anti-inflammatory pathway, thereby reducing pancreatic inflammation [18,25]. Nicotine also relaxes the Sphincter of Oddi dysfunction in experimental models, which reduces sphincter spasms and obstructions and may also have direct protective effects by reducing secretagogue-evoked cell necrosis in dispersed pancreatic acini [18,25]. Thus, we propose that the etiology of AP may have contributed to the heterogeneity. The RRs of ever smokers for both AP and CP were significantly heterogeneous, whereas for current and former smokers the heterogeneity was less remarkable or not present. This could be due to the inconsistent definition of ever smokers among the various articles we assessed and inconsistent reporting of the details of exposure. Another important source of heterogeneity was the contribution of dose differences. Therefore, we performed an analysis based on the cigarette consumption. However, data were only available for the AP group (Table 4). Most CP and pancreatitis studies did not specify the dose exposure differences.

As with all meta-analyses of observational studies, our results have certain limitations. First, the lack of uniformity in the categories of cigarette smoking was probably a weak point across the included studies, and certain smokers may have been misclassified because smoking exposure was not updated during the follow-ups. Second, a dose-response analysis could be conducted only for the AP group because exposure details were lacking for the other groups. Thus, we could only calculate the incremental changes in AP risk per 10 cigarettes smoked. Third, the majority of studies included in this meta-analysis were case-control studies, which are more susceptible to selection and recall bias. Fourth, the duration of smoking exposure should be considered as an important factor in the development of AP or CP. Consequently, the incidence of AP and CP as well as the strength of the correlation may have been underestimated because the duration of smoking and initiation of smoking varied considerably among studies. Moreover, there is concern that differences in country of origin, i.e., geographic differences, may have impacted the correlation between smoking and development of AP or CP. A recent prospective population-based study in Taiwan did not observe significant association between smoking and pancreatitis [26]. This supports significant differences between ethnic groups with respect to the risk of developing pancreatitis [26]. However, further exploration of potential ethnic differences is not feasible because of limited study in Asians, and thus more studies focusing on Asians are required.

In summary, the results from this meta-analysis suggest that cigarette smoking is associated with an increased risk of both AP and CP. Smoking cessation should be integrated into the management of patients with pancreatitis. Further studies, particularly of the dose-response relationship between cigarette smoking and pancreatitis, are required.

## Supporting Information

S1 PRISMA Checklist PRISMA Checklist for systematic review and meta-analysis. (DOC)

S1 Fig Cigarette smoking and the risk of acute pancreatitis without PEP. CI, confidence interval; RR, relative risk; PEP, post-endoscopic retrograde cholangiopancreatography pancreatitis.
(TIF)
