# Supplementary figures and images for: Impact of Smoking on the Risk of Pancreatitis: A Systematic Review and Meta-Analysis
Source: PLoS One. 2015 Apr 16;10(4):e0124075. doi: 10.1371/journal.pone.0124075 (PMC4399880; doi:10.1371/journal.pone.0124075)

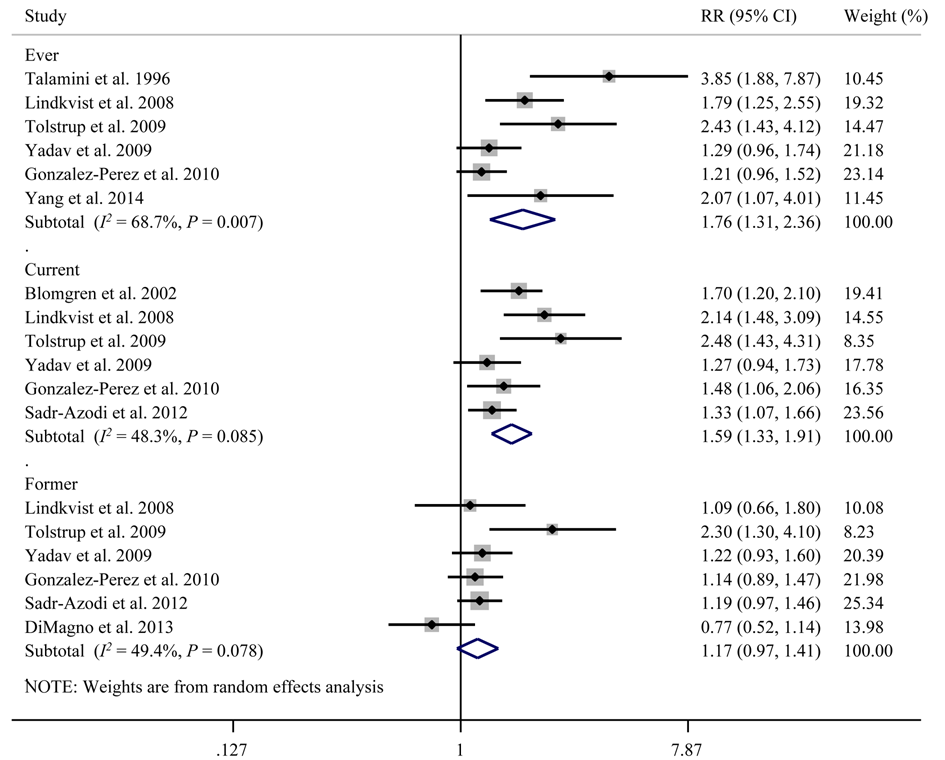

Supplement: S1 Fig — CI, confidence interval; RR, relative risk; PEP, post-endoscopic retrograde cholangiopancreatography pancreatitis. (TIF) [file pone.0124075.s002.tif]
